# Meeting the challenges posed by per diem in development projects in southern countries: a scoping review

**DOI:** 10.1186/s12992-020-00571-6

**Published:** 2020-05-28

**Authors:** Oumar Mallé Samb, Christiane Essombe, Valery Ridde

**Affiliations:** 1 Université du Québec en Abitibi-Témiscamingue 445 boul. de l’Université, Rouyn-Noranda, Québec J9X 5E4 Canada; 2 Université de Montréal 2900, boul. Édouard-Montpetit, Québec, Montréal, H3T 1J4 Canada; 3 CEPED (IRD-Université de Paris), Université de Paris, ERL INSERM SAGESUD, Paris, France

**Keywords:** Per diem, West Africa, Policy dialogue, Reform

## Abstract

**Purpose:**

This study presents the results of a review whose goal is to generate knowledge on the possible levers of action concerning per diem practices in southern countries in order to propose reforms to the existing schemes.

**Methodology:**

A synthesis of available knowledge was performed using scoping review methodology: a literature search was conducted using several databases (Medline, Cinahl, Embase, PubMed, Google Scholar, ProQuest) and grey literature. A total of 26 documents were included in the review. Furthermore, interviews were conducted with the authors of the selected articles to determine whether the proposed recommendations had been implemented and to identify any outcomes.

**Results:**

For the most part, the results of this review are recommendations supporting per diem reform. In terms of strategy, the recommendations call for a redefinition of per diems by limiting their appeal. Issued recommendations include reducing daily allowance rates, paying per diem only in exchange for actual work, increasing control mechanisms or harmonizing rates across organizations. In terms of operations, the recommendations call for the implementation of concrete actions to reduce instances of abuse, including not paying advances or introducing reasonable flat-rate per diem. That said, the authors contacted stated that few per diem reforms had been implemented as a result of the issued recommendations.

**Conclusion:**

The results of the study clearly identify possible levers of action. Such levers could make up the groundwork for further reflection on context and country-specific reforms that are carried out using a dynamic, participatory and consensual approach.

## Background

The Latin expression “per diem” (“per day” in English) refers to a daily allowance given to an individual/employee to cover expenses incurred as the result of an activity performed beyond his or her typical working conditions [1, 2]. This allowance is set by corporate-defined rates and requires no supporting documents, thus streamlining—in theory—administrative procedures by eliminating the various checks required for the reimbursement of expenses [1]. This practice is said to date back to the nineteenth century [1–3].

While the use of per diem systems is nothing new, the fact remains that the activities and work targeted by such allowances are often unclear and unremittingly spark controversy [4]. There seems to exist several types of jobs that warrant the use of per diem in the African context: 1) the reimbursement of an employee’s transportation costs and other related expenses incurred for work-related traveling; 2) capacity building through seminars, workshops and training; 3) financial incentives to increase employee satisfaction; 4) a means to simplify administrative processes allowing organizations to bypass the checks required in a reimbursement system based on actual costs [1–3, 5].

The perverse effects of per diem are also well known in the world of international development. In fact, the capacity building required for development gives rise to an increase in workshop and seminar opportunities whose per diem arouse more interest than the actual knowledge and abilities shared and delivered [1, 4–8]. As a result, seminar participants are often not the targeted individuals or those demonstrating the greatest interest in the subject at hand [1]. Instead, their participation is based on cronyism [9]. A study conducted in Burkina Faso showed the strategies of participants taking part in training programs who jump from one workshop to the next or who sign for absent colleagues to then split the per diem [5]. Smith frames such practices in a “workshop mentality” where events are organized without substantive content, for the sole purpose of receiving per diem allowances [8].

The perverse effects of per diem systems are also apparent on a macroscopic level. While it is difficult to quantify the scale of the problem because of a lack of related data and the cloak of secrecy [3, 10], the few available pieces of literature show that a significant portion of public expenses are claimed by per diems. For instance, a policy document prepared by the Public Policy Forum showed that in 2009 the Government of Tanzania spent US$390 million on per diems. This is the equivalent of the annual base salary of 109,000 teachers and 59% of the country’s payroll expenditures [2]. Furthermore, a study conducted in Tanzania by Soreide et al. found that 16.2% of the payroll (32% of the 2011 national budget) was allocated to the payment of daily allowances [3]. In Malawi, a 2010 study by Peprah and Mangani (cited by Skage) showed that staggering amounts were spent on travel-related per diems, representing 21.9% of wages [11].

In Burkina Faso, a study showed that 50% of the budget (approximately US$130,000), which was to be used in 2010 for planning the review of Burkina’s national sanitation policy and the drafting of the National Health Development Plan 2011–2020 (budget allotment: US$264,000), was spent on per diems for the various workshops. It should be noted that this does not include dinner breaks and other compensation for members of the technical drafting committee [5].

These examples give an indication of the extent of per diem-related spending. They also demonstrate how the matter of per diems can create a dependency on international aid to the point where State accountability is weakened with respect to their obligation to increase worker wages [12].

Familiar to and recognized by all, the issues related to per diem systems in LMIC (low- and middle-income countries) have led to a rise of related literature. Such literature includes scientific articles [8, 13, 14], professional commentaries [9, 15], news articles [16, 17] and even sharp criticisms made following an article addressing bureaucracy and the poor performance of certain governments [18]. No matter the definition, the incidents of abuse related to per diem take root in the paying out of sums to incite individuals to perform specific tasks in exchange for personal financial gain. Such practices demonstrating an unethical use of money have brought some individuals to draw a connection between per diem abuses and corruption.

[2, 12, 19]. Moreover, the practices related to hunting for per diem (also called “perdiemitis” [5]) have already pushed some authors to shine a light on research addressing the necessary adjustments generally needed to make less attractive the search for additional financial gain beyond typical salary. Back in 1997, the International Monetary Fund (IMF) reported that, in order to make public service wages as appealing as private sector or international NGO wages, on average, they would have to increase fivefold [7]. Another suggestion is to identify and encourage “positive deviance” [20], i.e., the development of qualified staff motivated by their work rather than the relentless pursuit of per diem [1].

However, can we implement these suggestions and are they real solutions? Compared to the literature aimed at exposing or condemning the problem, few studies have focused on possible solutions and reforms [2, 21]. As they would put it in political science [22], everyone is well aware of the situation. However, too few discussions are being organized to create windows of opportunity and seek relevant, viable and lasting solutions to these development ills.

Yet such information is required to reflect upon and formulate strategies on the different ways to solve this problem. Furthermore, such information would enable learning from the recommendations and initiatives that have been taken, while bridging the existing gaps [4]. This study outlines the findings of a scoping review whose goal is to map out the various reforms and proposed solutions to perdiemitis, along with the effectiveness of the solutions and the considerations needed for their implementation.

## Methodology

The main research question we wish to address is as follows: What makes up the current per diem reforms in LMIC and how effective are these reforms?

We used three specific questions to guide the literature search:

- What per diem reforms are currently taking place in LMIC?
- What aspects do they address and what are the contexts for implementation?
- What are the conditions for implementation and how effective are the reforms?

The knowledge synthesis was performed through a scoping review [23]—an exploratory synthesis. This method was chosen given the lack of previous reviews on the subject. A scoping review allowed to identify the presence and scope of existing literature, and to share the key messages. Our decision to pursue this type of review was also guided by the expected scarcity of related literature and our desire to map out the knowledge as well as the gaps.

The review was conducted in five steps [23]: 1) formulating a research question, 2) identifying studies and other relevant work; 3) selecting literature based on inclusion and exclusion criteria; 4) classifying data; and 5) gathering, synthesizing and reporting on findings.

Given the objective of mapping out existing knowledge, article methodology was not assessed. Accordingly, we used all the results meeting inclusion criteria. With the help of a librarian, three concepts and related keywords were chosen and used to perform the literary search. These concepts are as follows: 1) per diem; 2) reforms; and 3) southern countries.

Concept 1 refers to all the mechanisms leading to the payment of an amount beyond one’s wages, outside of the normal course of the employee’s duties.

Concept 2 refers to any change, restructuring or adjustment pertaining to the payment of an amount beyond set or suggested wages.

Concept 3 refers to LMIC as defined by the World Bank.

We adjusted the keywords of the second concept following an initial search cycle to eliminate any noise caused by its meaning when unaccompanied by other keywords and to pinpoint articles addressing reforms involving financial incentives. We paired keywords using Boolean and proximity operators. Truncations were also used (Table 1). We conducted a separate search for each concept, using keywords and descriptors proposed in the databases. We paired the results for each concept using the “AND” operator in order to perform a new search to find articles addressing all three concepts.

**Table 1:** Concepts, keywords and research strategies used in scientific databases

Concept 1	Per diem OR perdiem OR allowance* OR “daily allowance” OR “allowances paid” OR “allowance paid” OR “incentive program*” OR “financial incentive*” OR “compensation” OR “subsistence fee” OR subsistence* OR “subsistence allowance expense*” OR “subsistence allowance*” OR salar* OR “financial support” OR funding OR “living expense*” OR “living cost*” OR indemnit* OR “financial reward*” OR “rewarded financially” OR “financial counterpart*” OR “financial contribution” OR “allocating financial bonus*” OR “incentive plan*” OR “financial benefit*” OR “increased wage*”
Concept 2 (before and after adjustments)	Reform* OR leverage OR review* OR revision* OR control* OR ameliorat* OR amendment* OR amend* OR rearrangement* OR restructuring OR reorganisation OR reorganization OR overhaul OR reshuffle OR solution* OR improvement* OR agreement* OR suggestion* OR model*
	Reform* OR leverage OR revision* OR amend* OR rearrangement* OR restructur* OR reorganisation OR reorganization OR overhaul* OR reshuffle OR redesign* OR agreement* OR recast* OR reformulate OR rewriting
Concept 3	“Global South” OR “developing countr*” OR “low income countr*” OR “Middle income countr*” OR “Africa” OR “Sub Saharan Africa” OR “Latin America” OR “South America” OR “South Asia” OR “South East Asia” OR “Afghanistan” OR “Albania” OR “Algeria” OR “American Samoa” OR “Angola” OR “Argentina” OR “Armenia” OR “Azerbaijan” OR “Bangladesh” OR “Belarus” OR “Belize” OR “Benin” OR “Bhutan” OR “Bolivia” OR “Bosnia Herzegovina” OR “Botswana” OR “Brazil” OR “Bulgaria” OR “Burkina Faso” OR “Burundi” OR “Cabo Verde” OR “Cambodia” OR “Cameroon” OR “Central African Republic” OR “Chad” OR “China” OR “Colombia” OR “Comoros” OR “Congo” OR “Costa Rica” OR “Ivory Coast” OR “Cuba” OR “Djibouti” OR “Dominica” OR “Dominican Republic” OR “Ecuador” OR “Egypt” OR “El Salvador” OR “Eritrea” OR “Ethiopia” OR “Fiji” OR “Gabon” OR “Gambia” OR “Georgia” OR “Ghana” OR “Grenada” OR “Guatemala” OR “Guinea” OR “Guinea Bissau” OR “Guyana” OR “Haiti” OR “Honduras” OR “Hungary” OR “India” OR “Indonesia” OR “Iran” OR “Iraq” OR “Jamaica” OR “Jordan” OR “Kazakhstan” OR “Kenya” OR “Kiribati” OR “Korea” OR “Kosovo” OR “Kyrgyz” OR “Lao PDR” OR “Lebanon” OR “Lesotho” OR “Liberia” OR “Libya” OR “Macedonia” OR “Madagascar” OR “Malawi” OR “Malaysia” OR “Maldives” OR “Mali” OR “Marshall Islands” OR “Mauritania” OR “Mauritius” OR “Mexico” OR “Micronesia” OR “Moldova” OR “Mongolia” OR “Montenegro” OR “Morocco” OR “Mozambique” OR “Myanmar” OR “Namibia” OR “Nepal” OR “Nicaragua” OR “Niger” OR “Nigeria” OR “Pakistan” OR “Palau” OR “Panama” OR “Papua New Guinea” OR “Paraguay” OR “Peru” OR “Philippines” OR “Romania” OR “Rwanda” OR “Samoa” OR “Sao Tome” OR “Senegal” OR “Serbia” OR “Seychelles” OR “Sierra Leone” OR “Solomon Islands” OR “Somalia” OR “South Africa” OR “South Sudan” OR “Sri Lanka” OR “St. Lucia” OR “St Vincent the Grenadines” OR “Sudan” OR “Suriname” OR “Swaziland” OR “Syrian Arab Republic” OR “Tajikistan” OR “Tanzania” OR “Thailand” OR “Timor Leste” OR “Togo” OR “Tonga” OR “Tunisia” OR “Turkey” OR “Turkmenistan” OR “Tuvalu” OR “Uganda” OR “Ukraine” OR “Uzbekistan” OR “Vanuatu” OR “Venezuela” OR “Vietnam” OR “Gaza” OR “Yemen” OR “Zambia” OR “Zimbabwe”

The inclusion criteria for the articles included the following: 1) the article must address per diem as defined herein, and can use different terminology (for example: allowance, compensation); 2) the article must be set in a context of development projects (where development projects include various disciplines and initiatives aimed at improving the quality of life of populations) [24]; 3) the article must include a proposed or tested solution to per diem issues; and 4) the article must frame the problem and potential solutions within a LMIC, as defined by the World Bank. Articles referring to per diem issues without proposed solutions, and those addressing financial reforms on a macro level rather than within development projects, have been excluded.

To maximize the likelihood of getting results and gathering the viewpoints of various disciplines and areas of expertise, research was performed using scientific databases from several disciplines (health, political science, sociology, economics) and general databases (Additional file 1).

We also conducted a search for grey literature, i.e., “documents targeting a limited audience, outside commercial publication and distribution channels, and beyond the scope of bibliographic control devices” [25] [Loose translation]. The goal was to obtain documents from government and non-government organizations (Additional file 2), along with any related commentaries and suggestions by professionals working in development in southern countries. As the keywords and abstract of all scientific articles are in English, the research conducted in scientific databases was performed in English. The research conducted in grey literature databases was performed both in English and French.

The initial screening was performed using the article abstracts. The final selection was made following a full reading of the shortlisted articles. Furthermore, we contacted the authors of the selected articles in order to follow up on their recommendations. We asked them if there were any documents or information that could support the implementation data and speak to the responses and efficiency of the recommendations issued. We contacted the authors using the email addresses provided in the selected articles and we invited them to complete a five-question questionnaire (Additional file 3) or to take part in a Skype conversation. In August we contacted 20 authors, nine of which responded (45%): seven responded by email and two responded via Skype (Additional file 4).

The synthesis of results (obtained by reading the articles and exchanging with the authors) consisted in extracting data pertaining to the recommendations issued and to the context in which they were issued and/or implemented.

## Results

**Fig. 1:**
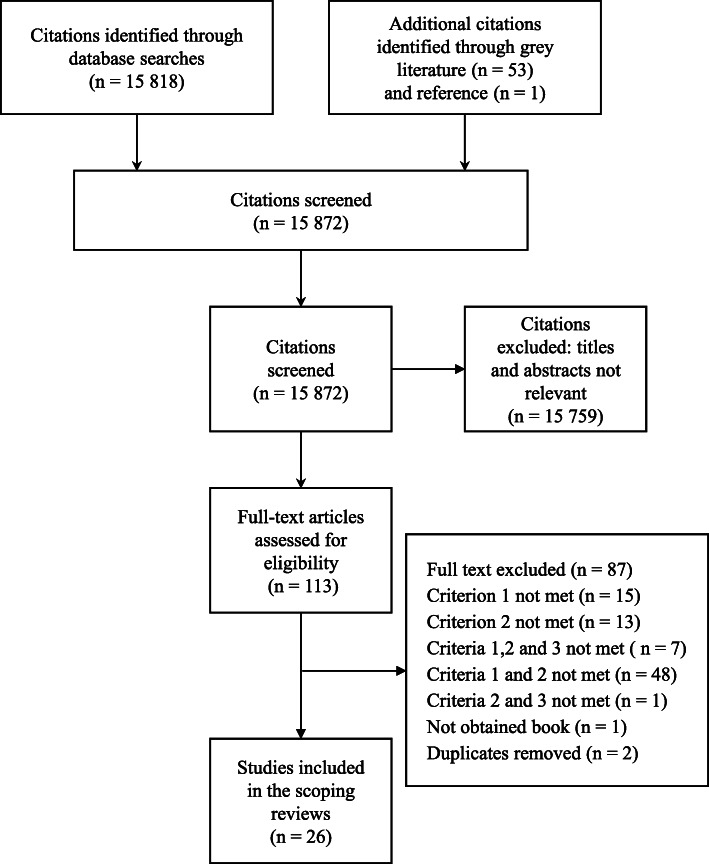
Prisma flow diagram

In total, we obtained 15,871 results following the database search, and one document was provided by a member of the research team. We analyzed 15,872 documents using their abstract and 113 articles through a full reading. Following the application of inclusion and exclusion criteria and the removal of any duplicates, we chose 26 documents (Fig. 1), including 9 scientific articles, 3 blogs or commentaries, 1 tool, 6 reports or policy briefs, 3 government documents, 1 editorial, 1 essay, 1 book chapter and 1 article from a journal (Additional file 5).

Most of the results from this synthesis are suggestions and recommendations for reform. We classified them according to their prevalence in the articles. It should be noted that only four published results [17, 19, 26, 27] are based on recommendations that have been effectively implemented.

### Recommendations

The recommendations issued in the articles spur per diem reform both on a macro level (policy recommendations) and on a micro level (operational recommendations). The policy recommendations call for a redefinition of per diem and of their terms of use so as to take into account other financial and social issues found in LMIC. The operational recommendations address practical issues related to their distribution (or non-distribution).

Such recommendations are as follows:

- Reform the per diem system by minimizing its appeal namely by reducing daily rates and always paying per diem in return for actual work [1, 9, 17, 19, 28, 28–31].
- Limit the use of per diem to the reimbursement of actual expenses for such things as traveling, and eliminate any further payments [3, 17, 19, 32, 33].
- Increase control mechanisms to limit abuse and sanction guilty individuals or organizations [2, 28, 34, 35].
- Harmonize per diem rates across organizations to avoid that individuals select workshops to attend according to the per diem rather than the topics discussed [9, 26, 35].
- Avoid paying advances, systematically require receipts and pay per diem only at the end of an activity, ideally by electronic payment rather than cash [17, 19, 33, 36].
- Perform research to identify the average local rates in order to implement a reasonable flat-rate per diem and/or prepay costly expenses such as staff accommodations [19].
- Transfer part or all the per diem to increase the wages of local staff where the project(s) is/are taking place [27, 37, 38].
- Develop indicators allowing to monitor the per diem allowances received by an employee [28].
- Conduct recurring internal and external audits and sanction fraudulent practices [28, 31].
- Establish development projects only with organizations possessing reliable accounting systems and refuse projects from organizations frequently accused of enabling per diem abuse [7, 28, 39].

### Implemented recommendations

From the nine authors who responded to our emails, the four authors contacted were unable to provide examples of implemented per diem reforms. Among these authors, two said that they could not guarantee what would become of their recommendations after they were issued and recognized a lack of leverage at their level to ensure action. One author mentioned a lack of funds and the fact that the topic had not been considered a priority when she sought to bring the issue before the development office in charge of financing the projects in which she had taken part in Tanzania, Malawi and Ethiopia.

Another author mentioned that, while he was no longer working on this topic, he believed major changes had taken place in Rwanda regarding per diem. The author also expected such changes to take place in Tanzania, without specifying which areas of development would be affected.

That said, the four authors contacted by email mentioned that they were aware of real-life cases in which per diem reform measures had been implemented following the issued recommendations. These include:

- One pilot intervention across three countries (Tanzania, Ghana and Burkina Faso) focused on testing different incentive models among healthcare staff [27]. Participants in the Nouna Department in Burkina Faso were the only ones to choose a model combining financial and non-financial incentives. However, the model has changed since the implementation of a performance-based funding program by the World Bank [40]. The two other countries chose non-financial incentives as a means of expressing their disapproval of any other incentives aimed at contributing fonds to per diem.

The elimination of flat-rate per diem paid before expenses are incurred and the provision of material or non-financial resources that can facilitate staff tasks; implemented by a leader of

- the Inter Aide Malawi programs, that are still in effect within this NGO [19] (Skype conversation with the author, August 25, 2016).
- A policy banning per diem by an organization overseeing a network of Scottish and Malawian NGOs [17] (Conversation with the author, August 26, 2016).
- Measures taken by the African Union (AU) and the European Union (EU). According to an author who served as mediator for the AU, such measures consisted in changing the AU’s per diem policy, including dropping the 20% top up for daily per diem allowances. The EU also made its funding to the AU conditional upon changes to the per diem policy. The AU is said to have consequently implemented a new per diem policy in 2014. The author did not, however, indicate the changes included within such policy. These recommendations are said to still be in effect, although met with great reluctance.

In light of the findings of this synthesis, it is possible to identify three separate levels of reform intervention: 1) a micro level made up of pilot projects; 2) a meso level in which per diem policies are implemented by agencies, NGOs and NGO groups, and applied with regard to all international partners; and 3) a macro level presented in this review through examples of reforms such as those within the AU and EU.

It should be noted that, to our knowledge, only one of these micro-level reforms (elimination of per diem within Inter Aide Malawi) has been assessed by an external advisor. Based on a review of the NGO’s financial statements and the comparison of expenses before and after the elimination of per diem, as well as each item of the child health program, the assessment concludes that Inter Aide made more efficient use of financial resources following the elimination of per diem. The advisor also concluded that Inter Aide Malawi made more efficient use of financial resources than other local NGOs (Skype conversation with the author, August 25, 2016). According to the advisor, these results are due to the fact that the elimination of per diem was conducted in parallel with the introduction of non-financial incentives such as the provision of equipment enabling enhanced working conditions.

Apart from this, we found no other formal assessment of these reforms. When we contacted the authors, we obtained only an appraisal of the efficiency of the reforms and the challenges related to their implementation.

Similarly to the NGO Inter Aide Malawi, which does not provide per diem but rather provides material assistance to facilitate project work (Skype conversation with the author, August 25, 2016), another example of an organization that does not provide per diem is Scotland Malawi Partnership [17]. This decision is based on regulations prescribed by the Scottish government and on the organization’s own position whereby per diem are a nuisance to development.

These two situations are examples in which the solution to per diem abuse is the elimination of per diem. In the first example, this decision emanates from a single person (Skype conversation with the author, August 25, 2016). In the second example, the decision to eliminate per diem is the result of a policy of the Scottish government and a corporate decision within the organization [17].

It should also be noted that in the case of Inter Aide Malawi, the decision to prohibit per diem was taken in the midst of a general policy review performed by the NGO itself.

According to Scotland Malawi Partnership, an umbrella organization encompassing several Scottish and Malawian organizations, the refusal of the Scottish government that funds allotted to NGOs be used to pay per diem and the organization’s policy banning per diem have not compromised project implementation. The author of the study claims that while some individuals have complained, the NGO has never been asked to substantiate its decision to other organizations.

or governments because they all understand the underlying reasons. Activities take place as planned and it is convened with partners from the very beginning that per diem are not provided (Skype conversation with the author, August 26, 2016).

Per diem policies cannot be established without consulting the other stakeholders involved [1, 15, 16]. Alignment across NGOs is therefore necessary, as stated by several authors [2, 7, 9, 17, 26, 28, 34, 39, 41].

Divergences exist not only across NGOs, but also between individuals. One of the authors with whom we spoke reported that upon presentation of his article and thoughts on strategies to avoid that the objectives of international meetings be superseded by per diem benefits, there was a heated debate between potential recipients (meeting participants) and donors (supranational unions, donor embassies). Also, several participants took the author aside to express their disagreement and discontent (email exchange with the author, August 2016).

## Discussion

### Reasons for inaction

An initial observation that was made is the scarcity of recommendations issued and/or followed to address issues related to per diem in southern countries. This is a perfect reflection of the muted response toward this well-known problem for which any solution would entail a change in the conceptualization of per diem and significant compromises that not all are prepared to make [42]. This can also be explained by the fact that not enough stakeholders holding levers of action see the problem as a priority, which would explain the relative scarcity of solutions being implemented [6]. In fact, while literature is scarce, the recommendations issued intersect and complement each other, thus showing the issue’s various levels of analysis, of which the authors are all well aware. That said, very few actions are taken.

The inaction on this issue is driven by two independent lines of thought that entertain different grounds of justification. On the one hand, an approach based on the institutionalization of a culture of per diem among national stakeholders who have long benefited from such practices and for whom per diem form an essential extrinsic motivation to promote implication in development projects [4]. As such, per diem are an opportunity that is to be seized as they offset what are considered poor wages [13, 43]. In fact, in fragile economies, per diem offered through development aid are seen by many as easy-access compensation goals that surpass State wages [1, 15].

On the other hand, an approach based on the internalization by donors of the requirement to provide per diem to avoid difficulties during project implementation [4, 44]. This second approach is the result of a historical weakness [1, 15, 45], path dependency [46]. This second approach obviously plays a decisive role in the current status quo which it works to protect by using per diem as ways to “buy the involvement” of local stakeholders whose presence can often be determined by looking at the per diem rates offered [44].

Consequently, per diem are stigmas of both weak economies and resource asymmetries between North and South which, ironically, are especially present within development projects aimed at reducing such resource gaps.

At any rate, the status quo supported by these two approaches cannot be sustained. It is based on a short-term vision [44] that will simply work on both sides to perpetuate this workshop mentality [8]. For instance, a study in Malawi demonstrates these challenges related to sustainability. Despite several years of efforts to strengthen the country’s information and health management system, the necessary monthly meetings to oversee progress became sporadic upon elimination of per diem [44].

The lack of implemented solutions also points to the bridge that is needed between assessments and compliance to issued recommendations [47]. Although many have spoken out about the problem, and some, reviewed herein, have issued suggestions, very few of these have been followed.

### Changing practices and mindsets: what are the levers of action?

The various underlying reasons explaining the appeal of per diem incite people to use them solely to reimburse actual expenses incurred for traveling or activities outside typical duties. The authors who have taken a firm stance against per diem in return for work have done so with the desire to leave unhindered the development process. Indeed, for those supporting this view, for the sake of sustainability, projects focused on promoting development should be delivered without financial compensation reserved for a select few based on their participation in a workshop that they might never actually use. The many workshops and trainings offering per diem and the lack of monitoring on knowledge that is reportedly acquired shape the ideal circumstances for such ills [5, 14].

Different approaches are suggested to avoid the complete elimination of per diem whose necessity has not been called into questioned. It is rather the approach chosen to obtain such top-ups that is perceived as non-ethical.

We thus observe three main trends to finding a middle ground between providing opportunities to increase wages and solving the epidemic of perdiemitis [5]. Firstly, we find initiatives aimed at promoting transparency and limiting abuse. Secondly, we find initiatives aimed at enabling the implementation of projects by providing financial incentives which enhance work conditions and having a positive impact on a larger scale than the few individuals who would receive per diem. Thirdly, we find initiatives aimed at enabling financial rewards based on performance. However, proof as to the efficiency of such initiatives has yet to be found [48, 49].

Initiatives focused on limiting instances of abuse seem to fit into a transactional approach in which the employee takes part in an activity and is then compensated for the costs incurred. Accordingly, the recommendations seek to limit the freedom to claim unnecessary expenses, suggesting that large expenses not be paid by the employee, that flat-rate sums supported by research on average local costs be used, and that receipts be demanded. The goal is thus to minimize the number of people attracted only by the related per diem and send a clear message about the existence of control measures.

Again with the goal of maximizing the number of employees seeking to serve a function rather than receive per diem, we find suggestions to award performance-based compensation and non-financial incentives to enhance performance. These suggestions serve a double purpose. First of all, the goal is to identity staff making good use of received training and material resources. These employees are rewarded with the opportunity to receive performance bonuses, i.e., wage top-ups based on merit. While this suggestion, in theory, allows for merit-based staff rewards, it is important to pair this initiative with a clear definition of performance, to ensure such definition is relevant, context-specific and verifiable in order to limit frustrations and fraud [50, 51]. Second of all, these suggestions promote skill building—a crucial part of any development project. These suggestions therefore prioritize instilling work ethic, which is often singled out as problematic in this situation [2].

Performing audits, encouraging the reporting of abuse, sanctioning all instances of fraud and promoting moral leadership—all listed in the findings—share the common goal of inspiring and rewarding moral practices in the workplace. However, one question remains. Does morality truly have its place in a difficult socio-economic environment where per diem abuse is driven by other economic structural shortcomings that have remained unsolved for several decades? The low number of recommendations implemented, the heated debates and the scant literature on the topic seem to indicate that the answer to this question is still at a very early stage. It will be difficult to implement recommendations as long as salary issues and the overall economic situation of the countries benefiting from development projects do not improve. Furthermore, the entrenchment and trivialization of questionable practices hamper initiatives seeking out change [6].

Routines, bad habits, simplicity, laziness or reluctance to tackle the issue. For some, complete elimination of per diem presents itself as no easy task. As an example, while the aforementioned Scottish government policy prohibiting per diem is formally respected by all government-funded Scottish NGOs, our contact suspects that some NGOs continue to pay per diem under other names. In fact, to ensure that their activities are not boycotted under the false pretence of being busy, as was found in Malawi [4], some NGOs have no other choice than to comply [4]. This, for instance, is the case in Senegal where the Groupe élargi des Partenaires Techniques et Financiers (called the G50) proposed in 2015 (final draft signed in December 2016) a complete overhaul of the per diem system in order to harmonize expenses for “local staff”. However, although the document was supported by the government, it is constantly causing tensions. For example, a union communication issued in early 2017 has led to many chief district physicians boycotting the meetings that apply the G50 protocol. The movement’s radical nature is such that some technical and financial partners have had to waive the application of the protocol in order to be able to conduct business. This was observed during a sharing workshop in which we participated and where the donor, to ensure the participation of the region’s chief physicians, contacted the union beforehand to guarantee that the G50 protocol, which it had signed, would not be in effect [52]. Moreover, in June of the same year, the Minister of Health requested the suspension of all workshops for a period of 2 months in order to avoid service disruptions. It would seem as though this decision was partly linked to the terms set by certain donors (including Japanese donors) for the draft and adoption of several policy documents in a timely manner to allow for the disbursement of significant funds, as the process was becoming rather lengthly.

### Reform through dialogue

To ensure their efficiency, the proposed reforms must be planned using a collaborative approach with the various stakeholders, and adapted to context-specific realities. Actual deliberative dialogues must be organized promptly [52, 53]. That said, few articles mention the involvement of potential per diem recipients in the drafting of recommendations. This review includes only four articles for which a discussion with potential per diem recipients was conducted [17, 19, 27, 54]. Incidentally, this is common practice in such contexts, where main recipients are often excluded from the reform crafting process [55]. Involving such stakeholders in the thought process is essential to support the emergence of solutions and the acceptance thereof [56].

## Conclusion

While there is abundant literature on issues related to per diem, literature addressing proposed solutions and reform mechanisms are scarcer. Data collected through our knowledge synthesis are, for the most part, suggestions. As for the very few initiatives that have been implemented, literature addressing their efficiency and necessary considerations for their implementation and monitoring are virtually non-existent. Therefore, the first conclusion of this synthesis is the existence of gaps in terms of proposed recommendations to tackle this important issue. The second conclusion is that there is more than one side to the problem, and each must be considered using a systemic approach. Basically, the recommendations tallied herein fall under three main categories: 1) transparency and limitation of abuse through the conceptualization of per diem as the reimbursement of expenses incurred; 2) promotion of optimal activity and project delivery through a supportive environment and work ethic; and 3) performance enhancement through non-financial resources and premiums. The complementary nature of the suggestions bears witness to the merit of a joint reflection addressing both prominent issues and underlying problems.

Lastly, it is important to note that, despite strong and widely differing positions regarding per diem, it is essential to bring together the most stakeholders as possible to increase the chances of arriving at a compromise deemed acceptable to the majority of stakeholders who will be in charge of its implementation. The tension and conflicts that typically feed these conversations [54] and the rare involvement of potential per diem recipients in such talks cannot be seen as a simple coincidence. Such conversations remain challenging as long as per diem are perceived as non-negotiable and irreplaceable. It is thus important for the various stakeholders involved in this problem to develop a peaceful framework for exchange, following the model of deliberative dialogues [52, 57]. This framework would have the complex task of representing different interests and viewpoints focused on recognizing the challenges leading up to the hunt for per diem, along with the its perverse effects and evidence.

One starting point for such reflections could focus on a strategy for a systematic approach to the problem by separating and prioritizing the various issues related to per diem, be it low wages, harmonizing per diem rates or targeting bad practices. Research and focus groups could be formed for each of these dimensions. Instead of searching for a one-size-fits-all solution, the goal would be to isolate the various drivers of per diem abuse and search for solutions to each of these drivers. This approach would allow to identify the common areas among the different issues raised and the proposed solutions. This way, solution-oriented research regarding per diem abuse could build on its own findings for certain specific aspects of the problem and see ongoing progress. This would also allow to broaden the scope and perform substantive work which would focus on the greater issues wherein lies the problem of per diem.

## Supplementary information

**Additional file 1.** Scientific databases.

**Additional file 2.** Governmental and non-governmental agencies and organizations of which a report or article written by an employee (current or former) is present or cited in the results.

**Additional file 3.** Questionnaire sent to the authors.

**Additional file 4.** Authors contacted and method of collecting data.

**Additional file 5.** Selected documents, issued recommendations and implemented recommendations.

## Data Availability

All data supporting our findings is contained in this articles and its supplementary information files.

## Abbreviations

LMIC: Low-and-middle-income countries
IMF: International Monetary Fund
NGO: Non-governmental organization
AU: African Union
ER: European Union
G50: Groupe élargi des partenaires techniques et financiers
