# Supplementary material for: Meeting the challenges posed by per diem in development projects in southern countries: a scoping review
Source: Global Health. 2020 May 28;16:48. doi: 10.1186/s12992-020-00571-6 (PMC7254660; doi:10.1186/s12992-020-00571-6)
Supplement: Supplementary file 1 — Additional file 1. Scientific databases. [file 12992_2020_571_MOESM1_ESM.pdf]

Additional file 1. Scientific databases

- CINAHL (EBSCO)
- Econlit (ProQuest)
- EMBASE (interface OVID, 1974–2015)
- Google Scholar
- International Bibliography of the Social Science (ProQuest)
- International Political Science Abstracts (EBSCO)
- International Relations (Oxford bibliographies)
- Ovid Medline
- PAIS index (ProQuest)
- Political Science (Oxford bibliographies)
- PubMed (NCBI interface, 1975–2015)
- Scopus
- Sociological Abstracts (ProQuest)
- Web of science.
- Worldwide Political Science Abstract (ProQuest)
