# Supplementary material for: Meeting the challenges posed by per diem in development projects in southern countries: a scoping review
Source: Global Health. 2020 May 28;16:48. doi: 10.1186/s12992-020-00571-6 (PMC7254660; doi:10.1186/s12992-020-00571-6)
Supplement: Supplementary file 2 — Additional file 2. Governmental and non-governmental agencies and organizations of which a report or article written by an employee (current or former) is present or cited in the results. [file 12992_2020_571_MOESM2_ESM.pdf]

Additional file 2. Governmental and non-governmental agencies and organizations of which a report or article written by an employee (current or former) is present or cited in the results

- African Development Bank
- Chr. Michelsen Institute (CMI)
- Communauté de l'Afrique de l'Est (East African Community)
- Inter Aide Malawi
- Groupe de coordination générale des partenaires techniques et financiers au Sénégal (G50).
- Norad (Norwegian Agency for Development Cooperation)
- République du Mali
- Scotland Malawi Partnership
