# Supplementary material for: Meeting the challenges posed by per diem in development projects in southern countries: a scoping review
Source: Global Health. 2020 May 28;16:48. doi: 10.1186/s12992-020-00571-6 (PMC7254660; doi:10.1186/s12992-020-00571-6)
Supplement: Supplementary file 3 — Additional file 3. Questionnaire sent to the authors. [file 12992_2020_571_MOESM3_ESM.pdf]

Additional file 3. Questionnaire sent to the authors

1. Connaissez-vous une (des) situation(s) où des recommandations (que vous avez formulées ou formulées par d’autres auteurs) visant à adresser les défis posés par les per diem, ont été mises en œuvre? O/N

2. Dans quel(s) cadre(s) ces recommandations ont-elles été mises en oeuvre? (Pays, nom de l’organisme, type d’activités pour lesquelles des per diem étaient octroyés, nombre approximatif de récipiendaires de per diem) Répondez « N/A » si vous avez répondu « Non » à la question 1.

3. Avez-vous connaissance de la réaction des récipiendaires de per diem suivant la mise en oeuvre de ces recommandations? Répondez « N/A » si vous avez répondu « Non » à la question 1.

4. Ces recommandations sont-elles toujours en place?

5. Existe-t-il des mesures ou indicateurs de leur efficacité? Si oui, pouvez-vous s’il-vous-plaît listez les mesures ou indicateurs dont vous avez connaissance?
