# Supplementary material for: Meeting the challenges posed by per diem in development projects in southern countries: a scoping review
Source: Global Health. 2020 May 28;16:48. doi: 10.1186/s12992-020-00571-6 (PMC7254660; doi:10.1186/s12992-020-00571-6)
Supplement: Supplementary file 4 — Additional file 4. Authors contacted and method of collecting data. [file 12992_2020_571_MOESM4_ESM.pdf]

Additional file 4. Authors contacted and method of collecting data

- Alexander Rowe: “The rise and fall of supervision in a project designed to strengthen integrated management of childhood illness in Benin” – Email
- Arne Tostenson: “When per diems take over: training and travel as extra” dans Corruption, Grabbing and Development: Real Word Challenges” - Ethiopia, Malawi, Tanzania – Email
- Arnold Peyrol Anglo: “Pour un système plus efficace de paiement des per diem en Afrique” – Email
- Piroska Bisits Bullen: “7 things you can do to help stop per diem abuse” - Malawi - Skype conversation
- Scotland Malawi Partnership (David Hope Johnson): « Practical advice on per diems » - Malawi – Skype conversation
- Stefan Hanson - “Need to reform the remuneration system to initiate a system approach to the health sector in resource-poor countries “- Email
- Taryn Vian: “Per diem policy analysis toolkit”- Ethiopia, Malawi, Tanzania - Email
- Thomas Tieku - “Perks Diplomacy: The Role of Perquisites in Mediation” - Email
- Maurice Ye: “Establishing sustainable performance-based incentive schemes: views of rural health workers from qualitative research in three sub-Saharan African countries” – Burkina Faso, Ghana, Tanzania – Email
