# Supplementary material for: Meeting the challenges posed by per diem in development projects in southern countries: a scoping review
Source: Global Health. 2020 May 28;16:48. doi: 10.1186/s12992-020-00571-6 (PMC7254660; doi:10.1186/s12992-020-00571-6)
Supplement: Supplementary file 5 — Additional file 5. Selected documents, issued recommendations and implemented recommendations. [file 12992_2020_571_MOESM5_ESM.pdf]

Additional file 5. Selected documents, issued recommendations and implemented recommendations

Author(s), title and type of document	Release date	Country	Issued recommendation(s)	Implementation of issued recommendations
Kremer, A. R.<br>"Pests and donors in Mali, 1985-1990" (30)<br>Scientific article	1992	Mali	Entrust the management of per diem to the leads of foreign partner agencies rather than delegate such procedures.	No
Pfeiffer J.<br>"International NGOs and primary health care in Mozambique: the need for a new model of collaboration"(37)<br>Scientific article	2003	Mozambique	Reduce or eliminate the instances of financial favors; create general NGO funds for the distribution of additional revenue to all health professionals from a district or province rather than only to employees working on a specific NGO project.	No
Republic of Mali<br>« Plan national d'action sur l'efficacité de l'aide au développement » (41)<br>National plan	2008	Mali	Align compensation for project and program staff services (per diem, salaries and benefits).	Yes?
Vian, T.<br>(CHR Michelsen Institute)<br>"Benefits and drawbacks of per diems do allowances distort good governance in the health sector?" (7)<br>Policy brief	2009	Cameroon, Malawi, Tanzania, Zambia	Discuss the value of per diem and per diem rates in relation to wages; encourage the coordination of donors, transparency and accountability.	No
Chêne, M.<br>"Low salaries, the culture of per diem and corruption" (9)<br>Expert response for U4	2009	Cambodia, Mozambique, Nigeria, Uganda, Tanzania, Zambia	Harmonize government and donor financial incentive regimes; promote a transparent, harmonized and accountable system that includes wage supplements; limit per diem payments to the reimbursement of actual expenses—not seminar participation; offer standard rate per diem; develop two separate standards for the payment of wage supplements and financial incentives; require that donors provide the government and donors with the names of wage supplement and/or monetary incentive recipients; sign and comply with per diem policies.	No
Jansen, E. G.<br>(CHR Michelsen Institute)<br>"Does aid work? Reflexion on a natural resource program in Tanzania" (39)<br>Report	2009	Tanzania	Strengthen the financial management system of aid programs; have Department staff trained by independent foreign accountants who are authorized to monitor accounts; closely monitor the flow of program funds; never sign an agreement for a new program before the implementation of a satisfactory financial management system; prioritize the various dimensions of proper governance.	No
Jack, A<br>"Expenses culture has high cost for world's poorest nations" (16)<br>Journal article	2009	Non-specified; general thoughts on the issue	Pay per diem outside government guidelines by using rewards based on individual performance.	No

Peters, B.<br>"Per diem: To pay or not to pay? That is the question" (12)<br>Commentary	2010	Ethiopia	Reimburse actual expenses for travel, accommodations and meals with supporting receipts; reassess the current flexibility for discretionary expenses and estimates of required amounts for international expenses.	No
Rowe, A., Onikpo, F., Lama, M. et Deming, M.<br>"The rise and fall of supervision in a project designed to strengthen integrated management of childhood illness in Benin" (29)<br>Scientific article	2010	Benin	Leverage resources and supervisor authority to promote surveillance (e.g., prohibit per diem admissible non-surveillance activities during surveillance periods).	No
Chan, H.S. et Ma, J.<br>"How are they paid? A study of civil service pay of China" (31)<br>Scientific article	2011	China	Eliminate non-wage sources of income; strengthen budget controls through budgetary reforms by consolidating extra-budgetary funds; streamline the payroll system.	Yes
Vian, T. et Sabin, L.<br>(CHR Michelsen Institute)<br>"Per diem policy analysis toolkit" (34)<br>Toolkit	2012	Ethiopia, Malawi, Tanzania	Reduce pressures leading to the hunt for per diem; control discretionary measures, increase transparency and accountability; enhance fraud detection and control implementation; support leaders.	No
Søreide, T., Tostensen, A. and Ingvild, A. S.<br>(Norad Evaluation Department)<br>"Hunting for per diem: The uses and abuses of travel compensation in three countries" (3)<br>Report	2012	Ethiopia, Malawi, Tanzania	Use per diem to reimburse travel expenses only; promote enhanced control systems; guarantee the value of sums invested in training and activities with per diem expenses; meet the challenges faced by donors.	No
Martinez, P., Rajabo, M., Bambo, C., Smith, M., Negrete, I., Augusto, G. et al.<br>"Strengthening the competencies of mid-level clinicians during pre-service training to improve the care and treatment of HIV+ patients in Mozambique" (38)<br>Scientific article	2012	Mozambique	Strengthen the skills of clinicians prior to their postings in hospitals to reduce training and per diem expenses afterwards.	No
Hanson, S.<br>"Need to reform the remuneration system to initiate a system approach to the health sector in resource-poor countries" (32)<br>Editorial	2012	Reflexion on the issue in sub-Saharan Africa, Tanzania and East Africa, as mentioned.	Link compensation and per diem to costs incurred; deliver wages regularly and "fairly" by combining long and short-term performance-based bonuses.	No

Vian T, Miller C, Themba Z et Bukuluki P.<br>“Perceptions of per diem in the health sector: evidence and implications” (2)<br>Scientific article	2013	Malawi, Uganda	Reduce pressure and opportunities of abuse while promoting accountability and transparency; develop policy, control and assessment analysis tools; increase worker wages and deliver timely payment of wages; harmonize per diem rates across organizations; enhance monitoring, complaint mechanisms and policy implementation; develop better planning (e.g., plan one workshop at a time) and a better use of technology; promote integrity; support frequent staff transfers to avoid the entrenchment of bad practices; enhance dialogue without enhancing incentives; align the roles and contributions of NGOs and development partners with government institutions; support baseline data collection and assess pilot interventions for per diem.	No
Tieku T.K.<br>“Perks Diplomacy: The Role of Perquisites in Mediation” (54)<br>Scientific article	2013	Based on the Peace Negotiations in Burundi, held in Arusha, Tanzania from 1996 to 2000 Seventh round of inter-Soudanese Peace Talks in Abuja, Nigeria from 2005 to 2006	Use the Mandela model: Hold talks and negotiations in houses rather than hotels; offer guests the same compensation and conditions as those of the host country’s public servants; adjust the AJQ rates to match those of the host country’s public servants.	No, however, the article has led to the implementation of other reforms: the AU has dropped the extra 20% from its daily allowances and the EU has made its financing of the AU conditional upon changes to the per diem policy.
Scotland Malawi Partnership<br>« Practical advice on per diem » (17)<br>Report	2014	Malawi	Avoid paying per diem; pay only expenses incurred; discuss the per diem policy with partner; deliver only the meetings and workshops that are critical for those participating; provide travel allowances and accommodations.	Yes
Skage I, Søreide T et Tostensen A.<br>“When per diem take over: training and travel as extra” dans Corruption, Grabbing and Development: Real Word Challenges.” (11)<br>Book chapter	2014	Ethiopia, Malawi, Tanzania	Enhance the monitoring of seminar and workshop organization; combine a control system with performance recognition; assess the impacts of training programs on service enhancement; clarify that compensation systems are to be used only for the reimbursement of expenses; teach supervisors the proper use of per diem; prohibit daily allowance inflation to attract new participants; ensure that training programs are subject to independent audits.	No
Nkamleu G. B. et Kamgnia B. D.<br>“Uses and Abuses of Per-diem in Africa: A Political Economy of Travel Allowances” (1)<br>Working paper	2014	Malawi, Nigeria, Tanzania	Reconceptualize the function of per diem with a focus on the reimbursement of expenses; find a different way to increase staff motivation; reduce daily per diem rates.	No
Yé, M., Aninanya, G. A., Sié, A., Kakoko, D. C. V., Chatio, S., Kagoné, M. et al.<br>“Establishing sustainable performance-based incentive schemes: views of rural health workers from qualitative research in three sub-Saharan African countries” (27)<br>Scientific article	2014	Burkina Faso, Ghana, Tanzania	Promote non-financial incentives; direct a part of per diem toward mutual funds for all members of staff; provide team incentives rather than individual incentives.	Yes, however, the initiative has been replaced by another program.

UK-Essay (unknown author)<br>"Low wages and corruption in Nigerian public service " (35)<br>Essay	2015	Nigeria	Reform the incentive structure of the public sector and financial aid scheme; deliver efficient fraud control; limit training opportunities with per diem; harmonize donor per diem.	No
Skage I, Søreideb T et Tostensenc A.<br>"Carpe Per Diem: The Uses and Abuses of Travel Compensation in Developing Countries" (28)<br>Scientific article	2015	Ethiopia, Malawi, Tanzania	Improve the revenue audits of State institutions; reform salaries; enhance controls over a short period of time to break bad habits; show transparency regarding budgets and national audit results; ensure the independence of the National Audit Office; request project and program audits; avoid perceiving skill-building activities as performance indicators; avoid facilitating the discretionary delivery of per diem; facilitate transparency in per diem rate selection procedures; harmonize per diem rates and systems across donor communities and government bodies; secure incident reporting systems.	No
Groupe de coordination générale des partenaires techniques et financiers au Sénégal (G50).<br>Protocole d'accord sur l'harmonisation des coûts locaux appliqués au personnel local (gouvernement, société civile) (26)<br>Memorandum of understanding	2015	Senegal	Harmonize per diem and travel expense amounts; cover only reimbursement fees; pay providers directly when space is not provided free of charge; never pay government officials acting as facilitators, moderators or reporters; never reimburse travel expenses for citizens of the place where the activity is being held.	Yes
Republic of Mali<br>«Accord sur les frais et indemnités dans le cadre des programmes et projets financés par l'aide extérieure » (33)<br>Draft agreement	2016	Mali	Mission and activity participation expenses must be in consideration of the actual expenses incurred; send provider invoices directly to technical and financial partners and NGOs; substantiate per diem payments; pay flat-rate accommodation, travel and meal allowances.	To be confirmed
Anglo, A.<br>"Pour un système plus efficace de paiement des per diem en Afrique" (36)<br>Blog	2016	Non-specified; general thoughts on the issue in sub-Saharan Africa	Use per diem to cover mission officer expenses; develop a reimbursement table with smaller amounts; provide supporting documents; specify the rates of pay indistinctly of hierarchy through an enquiry; reimburse at the end of the activity only and following presentation of supporting documents; make payments through bank transactions; revalue wages; create planning tools and decrease the number of meetings/activities.	No
Piroska Bisits, B.<br>"7 things you can do to help stop per diem abuse" (19)<br>Blog	N/A	Malawi	Set per diem rates according to average local rates; offer the same per diem independently of position; use per diem for travel expenses only; make reservations for staff and limit workshops in terms of quantity and number of participants.	Yes
